# Robust and Easy-to-Use One-Pot Workflow for Label-Free Single-Cell Proteomics

**DOI:** 10.1021/acs.analchem.2c05022

**Published:** 2023-02-20

**Authors:** Manuel Matzinger, Elisabeth Müller, Gerhard Dürnberger, Peter Pichler, Karl Mechtler

**Affiliations:** † Institute of Molecular Pathology (IMP), Campus-Vienna-Biocenter 1, 1030 Vienna, Austria; ‡ Institute of Molecular Biotechnology of the Austrian Academy of Sciences, Dr. Bohrgasse 3, 1030 Vienna, Austria; § Gregor Mendel Institute of Molecular Plant Biology (GMI) of the Austrian Academy of Sciences, Dr. Bohrgasse 3, 1030 Vienna, Austria

## Abstract

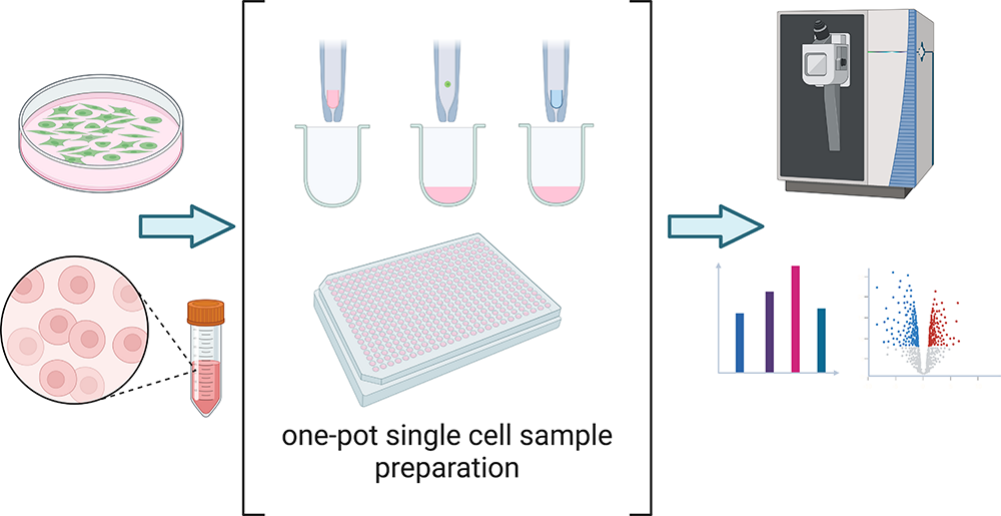

The analysis of ultralow input samples or even individual cells is essential to answering a multitude of biomedical questions, but current proteomic workflows are limited in their sensitivity and reproducibility. Here, we report a comprehensive workflow that includes improved strategies for all steps, from cell lysis to data analysis. Thanks to convenient-to-handle 1 μL sample volume and standardized 384-well plates, the workflow is easy for even novice users to implement. At the same time, it can be performed semi-automatized using CellenONE, which allows for the highest reproducibility. To achieve high throughput, ultrashort gradient lengths down to 5 min were tested using advanced μ-pillar columns. Data-dependent acquisition (DDA), wide-window acquisition (WWA), data-independent acquisition (DIA), and commonly used advanced data analysis algorithms were benchmarked. Using DDA, 1790 proteins covering a dynamic range of four orders of magnitude were identified in a single cell. Using DIA, proteome coverage increased to more than 2200 proteins identified from single-cell level input in a 20 min active gradient. The workflow enabled differentiation of two cell lines, demonstrating its suitability to cellular heterogeneity determination.

## Introduction

The investigation of individual cells using advanced technologies for cell isolation and analysis of their nucleotide content has revolutionized the current depth of knowledge in the areas of cellular development and behavior.^1−4^ However, cellular identity is driven by its proteome, which is why proteome profiling has become increasingly popular in molecular cell biology research. Reaching single-cell resolution is important because subtle changes in protein expression and turnover can lead to changes in cellular behavior and function. Even clonally identical cells differ from each other, depending on their environment and age. Understanding cellular heterogeneity is therefore important to understanding regulatory mechanisms and the development of cells such as stem cells.

To obtain a comprehensive and quantitative proteome profile by classical mass spectrometric techniques requires the bulk analysis of tens of thousands to millions of cells.^5^ Biochemical approaches such as flow cytometry, immunoprecipitations, or immunofluorescence-based techniques help to resolve intercellular networks but are limited in specificity to approximately a dozen defined proteins.

Single-cell proteomics aims to fill this gap by resolving cellular heterogeneity on global and unbiased levels.^6^ With attomolar detection limits, there is no doubt that mass spectrometry (MS) is sensitive enough to perform this task. The median number of protein copies in an individual mammalian cell is estimated to be 18,000,^7^ meaning that MS identification of at least the most abundant fraction of proteins in a cell is possible. However, reaching sufficient proteome depth remains challenging due to workflow limitations, including:

- Sample losses: Due to contact with surfaces and purification processes, the manipulation of cellular content during sample preparation and its transfer into a mass spectrometer can result in losses, leading to significantly lower workflow sensitivity and reproducibility. Techniques recently developed such as nanoPOTS^8,9^ or the proteoCHIP^10^ minimize sample losses by minimizing handling volumes and automating sample handling, but require specialized equipment.
- Sensitivity: Sample separation and detection of ions in the mass spectrometer must be fine-tuned and often lack sufficient sensitivity.
- Throughput: To enable definitive statistics, hundreds to thousands of cells must be analyzed. To increase sensitivity and throughput, multiplexed analysis and carrier proteomes are commonly used in single-cell proteomics workflows.^9−13^ However, these approaches reduce quantitative accuracy and dynamic range compared to label-free approaches, and the choice of carrier proteome potentially biases the type of peptides identified.^14−16^

Here, we present a single-cell analysis workflow that provides a complete set of fine-tuned steps from sample preparation through data analysis. The workflow addresses the limitations associated with current approaches such as using standard 384-well plates used only in few other protocols,^11,17,18^ which makes it adoptable by a broader community of scientists. Further, the workflow enables the use of shorter gradients without loss of separating power, increasing throughput and allowing more robust label-free quantitation (LFQ) strategies. Finally, advanced data acquisition strategies are combined with AI-based data analysis to increase identifications (IDs) and dynamic range.

## Results

### Optimizing Cell Isolation and Digestion to Enhance Recovery and Reproducibility

**Figure 1:**
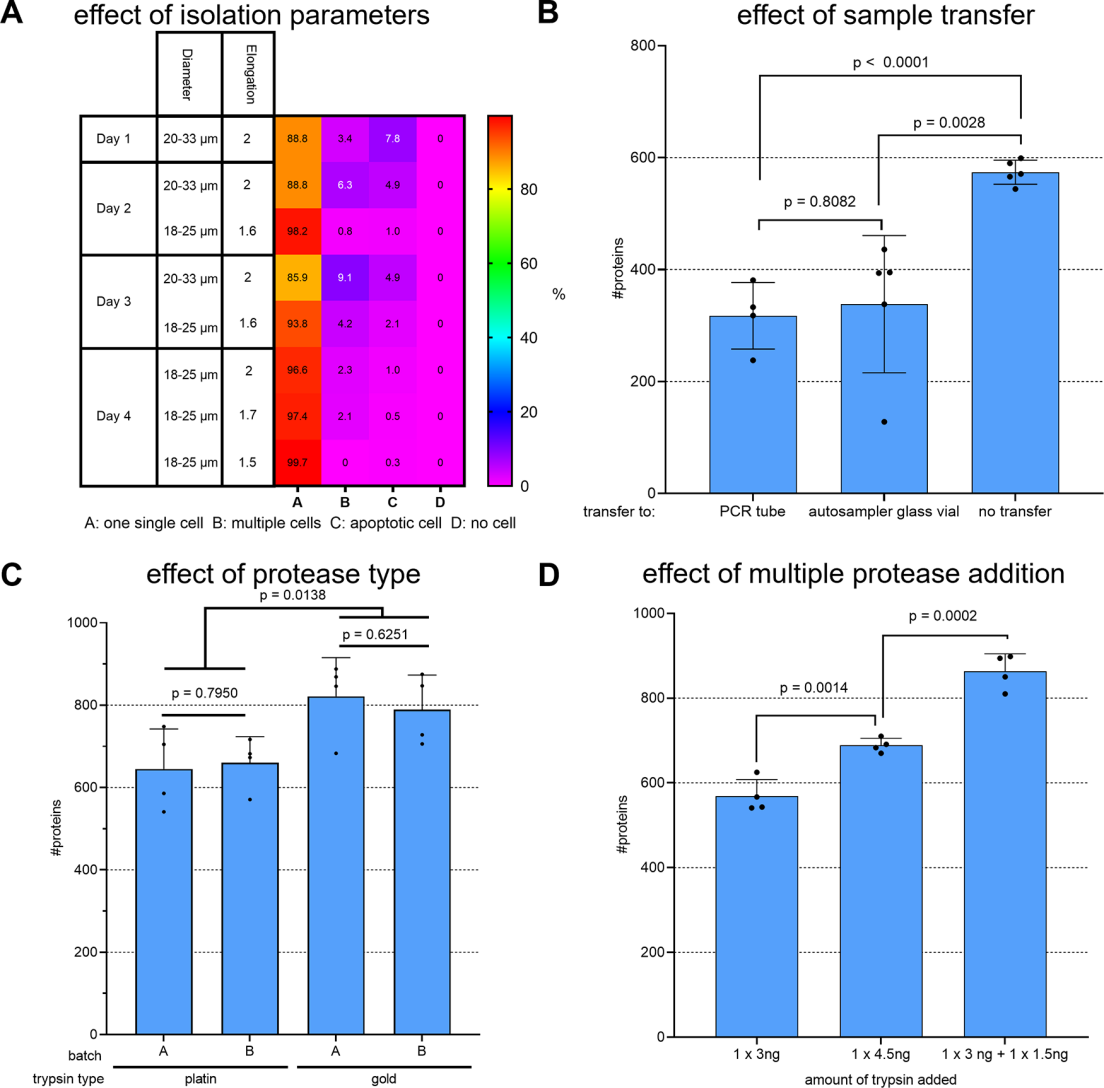
Improving recovery and reproducibility by tuning workflow parameters. Individual HeLa cells were isolated into a predispensed master mix using CellenONE. (A) Parameters diameter and elongation were varied on CellenONE; numbers indicate the relative amount (%) of cells isolated in the category as indicated, *n* = 384 cells on 4 individual days each. (B) Individual HeLa cells were lysed and digested in a 384-well plate, and the sample was either injected from there to the LC-MS system or transferred as indicated (1 × 3 ng + 1 × 1.5 ng trypsin added, no DMSO was supplemented). (C) Cells were digested with two different batches of trypsin gold or platinum, each as indicated (1 × 3 ng + 1 × 1.5 ng trypsin added, no sample transfer, 5% DMSO supplemented). (D) Trypsin was added either once within the master mix (3 ng in 1 μL) or a second time by addition of 500 nL of additional fresh 3 ng/μL trypsin after 30 min. For comparison, the same total amount (4.5 ng in 1μL, middle bar) added in the beginning was included as the experimental condition (all conditions: without sample transfer and with 5% DMSO supplemented) (B–D) DDA data was analyzed using CHIMERYS at 1% FDR. The bars and error bars show the protein IDs obtained with their standard deviations. An unpaired two-tailed Student’s *t*-test was performed, and the resulting p-values are depicted above each compared data set, *n* ≥ 4 replicates.

Aiming for full workflow automatization, ideal cell isolation parameters must be chosen. When using the cellenONE robot (Cellenion), diameter and elongation are the relevant parameters. For this study, these parameters were initially optimized for the selection of individual HeLa cells. To estimate the best set of initial parameters, the distribution of all detected cells was mapped. In line with our expectations,^19^ the majority of cells had diameters ranging from 15 to 30 μm. As a first reference, we chose the standard parameters used in our lab before, for the isolation of HeLa cells. We isolated 384 single cells into a 384-well plate. Visual analysis of the images of each isolated cell revealed four different cases: only one cell was isolated per well, more than one cell was isolated per well, the isolated cell appeared apoptotic, or no cell was isolated at all (Figure 1A). Supporting Information Figure 1A–D shows representative examples of each case. Overall, only 88.8% of all wells confidently contained exactly one intact cell. Reducing the diameter range and elongation to a lower value ensured that multiple attached cells were not falsely recognized as single cells and that only intact living cells were isolated by a stricter elongation parameter. Using parameter optimization, the fraction of correctly isolated individual intact cells was eventually increased to 99.7%, which allowed for automation of cell isolation without manually and visually validating images of each isolated cell. To validate selected parameters, condensed DNA of apoptotic cells was stained with Hoechst 33342 solution^20^ (Thermo Scientific Pierce), confirming exclusive selection of viable cells (Supporting Information Figure 1E).

For label-free single-cell sample preparation, a protocol based on the work of Ctortecka et al.^10^ was adopted. Briefly, 1 μL of master mix containing the detergent DDM for lysis, the enzyme trypsin for digestion, and TEAB buffer was predispensed to each well of a 384-well plate, followed by isolation of individual HeLa cells into this master mix (for details, see the Methods Section). After that, lysis and digestion start with incubation inside the cellenONE for 2 h at 50 °C and 85% relative humidity. Since sample transfers are prone to loss of hydrophobic peptides, transfer to different tube materials for storage was compared to sample storage and injection directly from the same 384-well plate also used for sample preparation (Figure 1B). Both transfer to a PEG-coated PCR tube, as described by Ctortecka et al.,^10^ as well as transfer to a glass vial, lead to obvious losses compared to omitting any sample transfer. In conclusion, all future single-cell samples were processed in and injected from a 384-well plate. Next, the effect of the used protease (Figure 1C) as well as of multiple protease addition (Figure 1D) was assessed. In detail, trypsin platinum and gold (both Promega) were compared. According to the manufacturer, trypsin platinum is free of any detectable nonspecific proteolytic activity, which is why an improved overall digestion efficiency is to be expected. Surprisingly, trypsin gold delivered slightly better results in our single-cell setting in terms of both protein identifications and observed missed cleavages. Because the tryptic activity is expected to decrease with time,^21^ trypsin was added again after 30 min, which significantly increased the number of identified proteins compared to using a lowered or the same total amount in a single addition (Figure 1D). Additionally, we tested the protease enhancer ProteaseMAX from Promega. On average, we were able to achieve a 17% increase in the proteins found in the single-cell samples by adding the enhancer to our trypsin digestion (Supporting Figure 4). Therefore, all subsequent experiments were performed by adding ProteaseMAX to the master mix and Trypsin Gold twice. The detailed method steps used to perform the cell isolation, sample transfer, and digestion experiments are provided in the Supporting Information.

### Optimizing Sample Preparation to Boost Protein IDs

**Figure 2:**
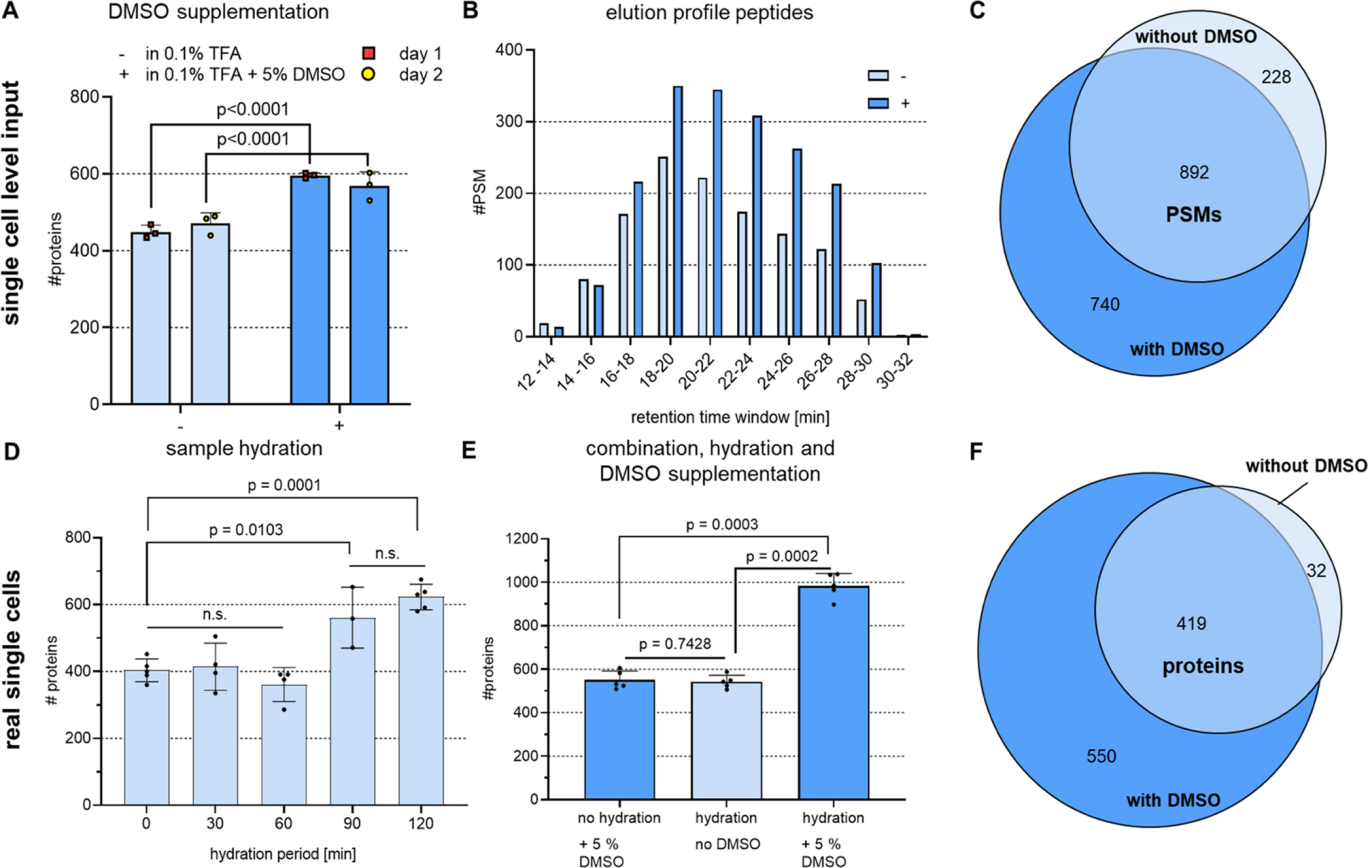
DMSO supplementation and sample hydration improved the recovery of hydrophobic peptides and boosted IDs. All data were acquired using DDA and then analyzed using the CHIMERYS algorithm with 1% FDR at the PSM and protein levels. (A) 250 pg HeLa bulk digest dissolved at a concentration of 71 ng/μL in the presence or absence of 5% DMSO prior to injection into the LC-MS. The bars and error bars show the resulting protein IDs and their standard deviations. To estimate significance, an unpaired two-tailed Student’s *t*-test was performed. The resulting *p* values are shown above each compared data set, *n* = 6 technical replicates measured on 2 different days. (B) Data from a representative replicate of the DMSO tests performed in panel (A). The bars present the PSM matches in each retention time window. (C) Venn diagram showing the common PSMs from the protein IDs shown in panel (B). (D) Individual HeLa cells were lysed and digested at 50 °C for 2 h, while the sample was hydrated with automated water supplementation for the time periods shown. The bars and error bars show the resulting protein IDs and their standard deviations. A one-way ANOVA with Tukey’s multiple comparison post-test was performed, *n* ≥ 3 replicates. (E) Individual HeLa cells were kept hydrated for 2 h or dried upon evaporation during digestion in the presence or absence of DMSO in the final storage solution. The bars and error bars show the resulting protein IDs with their standard deviations. An unpaired two-tailed Student’s *t*-test was performed, and the resulting *p* values are shown above each compared data set, *n* = 5 replicates. (F) Venn diagram showing the common protein IDs from a representative replicate comparing hydration and no DMSO with hydration plus 5% DMSO from the data shown in panel (E).

Though using 1 μL instead of nanoliter-range volumes makes sample handling more convenient, it increases wetted surface area and the chance that peptides will stick to the tube walls. With this in mind, we attempted to keep peptides solubilized by supplementing 5% DMSO. Use of DMSO was tested on diluted bulk digests at a concentration of 250 pg/3.5 μL, which was the same as the concentration expected in the real single-cell samples under ideal conditions. As demonstrated by the additional protein IDs obtained, DMSO reproducibly and significantly increased recovery (Figure 2A). The additional peptides found in the DMSO-containing samples predominantly eluted toward the end of the gradient (Figure 2B), supporting the conclusion that DMSO supplementation improves the solubility of hydrophobic peptides. In addition, the vast majority of 80% of all peptide-spectrum matches (PSMs) found in a representative replicate analysis performed without using DMSO were ultimately found when DMSO was added (Figure 2C). Those not found could be due to the inherent run-to-run variability of the stochastic DDA method used.

To minimize adsorptive sample losses also during single-cell sample preparation, we further aimed to hinder sample drying by automated addition of water during digestion at 50 °C within CellenONE. By that, the digestion mix was kept hydrated over a period from 0–120 min until the end of incubation. In the case of no hydration task performed, the sample was reproducibly dried after ∼30 min, which is why 500 nL of water was added every 15 min to keep the volume at the same level. Our results suggest that elongated hydration allows for longer active digestion and lowered sample adsorptive sample loss, as indicated by more proteins identified when hydrating at least over the first 90 min of the digestion process (Figure 2D). Analyzing single cells that were either kept hydrated during digestion or supplemented with DMSO for storage led to roughly 500 protein IDs. Combining both boosting strategies results in more than 1000 average proteins identified from a single HeLa cell using DDA and analyzing data with CHIMERYS on 1% FDR at peptide and protein level (Figure 2E). This effect is even more distinct on the peptide level, where an average of 1883 peptides was found without DMSO of hydration compared to 4625 with hydration and DMSO, which corresponds to a boost by 146%. Notably, adding DMSO enabled identification of 550 additional proteins, with 93% of all proteins previously identified in the absence of DMSO found (Figure 2F). All data were acquired using DDA and then analyzed using the CHIMERYS intelligent search algorithm (MSAID), with 1% FDR at the PSM and protein levels. The detailed method steps used to perform the sample preparation experiments are provided in the Supporting Information.

### Sample Preparation Workflow Key Points

**Figure 3:**
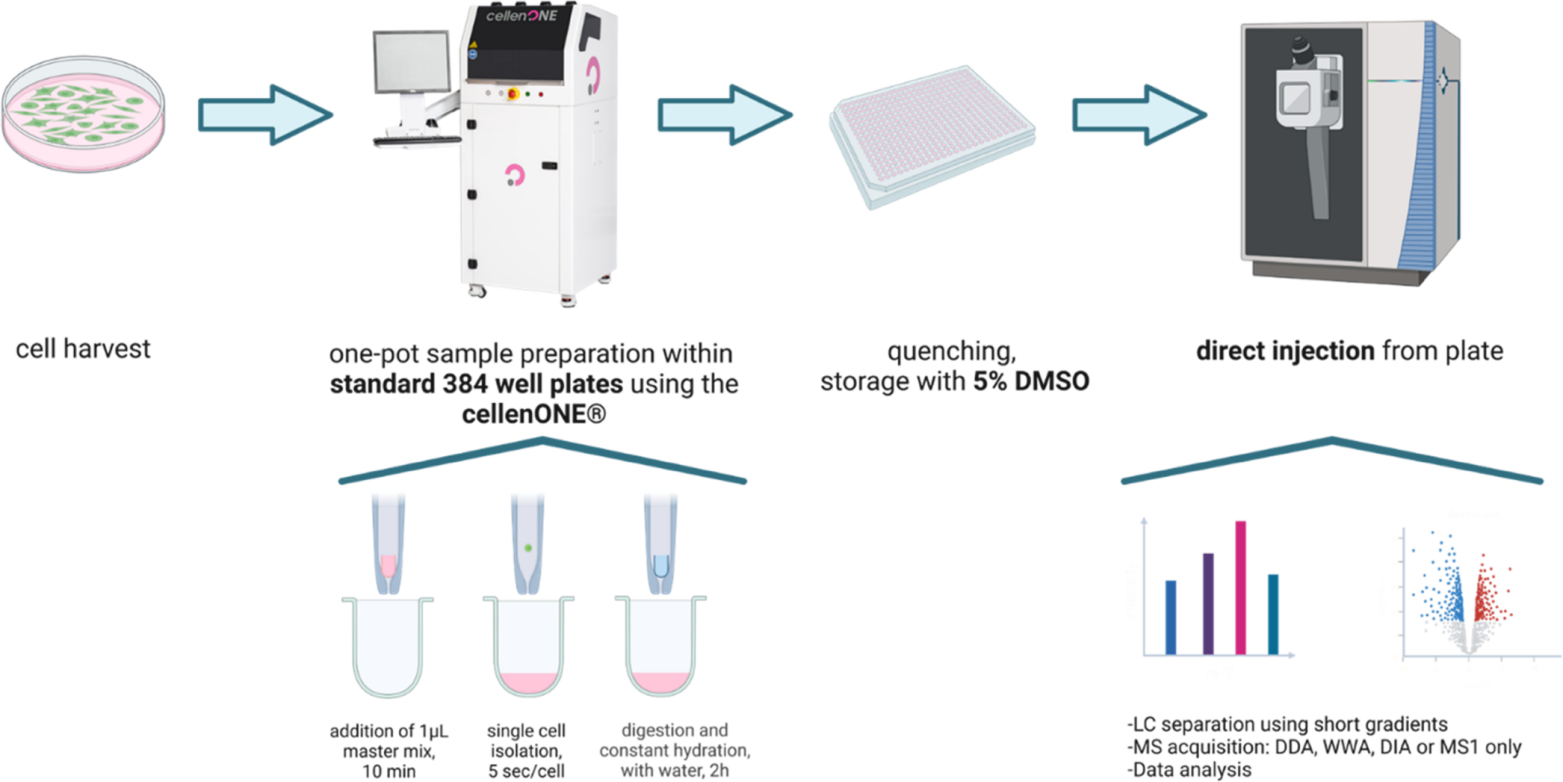
Overview of label-free, one-pot, single-cell sample processing workflow. Cells are harvested, followed by lysis and digestion within CellenONE in a 384-well plate that is also used for direct placement into the autosampler of the HPLC-MS. Figure created with BioRender.

To ensure a robust, reproducible, and automated single-cell workflow, single-cell isolation was performed using the cellenONE picolitre dispensing robot. Using a 384-well plate and easy-to-handle 1 μL volumes allows laboratories to implement the workflow without the need for expensive and specialized ultralow-flow equipment and therefore also in combination with other cell isolation machines as a FACS device commonly available in many lab environments. Constant hydration was automatically performed by cellenONE but can be replaced by manual pipetting. The same is true for providing 1 μL of master mix in each well, which can be done faster and without change in overall performance using a multichannel pipette (see Supporting Information Figure 8). Our final workflow is sketched in Figure 3 and detailed within the methods section (Supporting Information).

### Optimizing LC-MS Data Acquisition and Analysis to Maximize IDs

**Figure 4:**
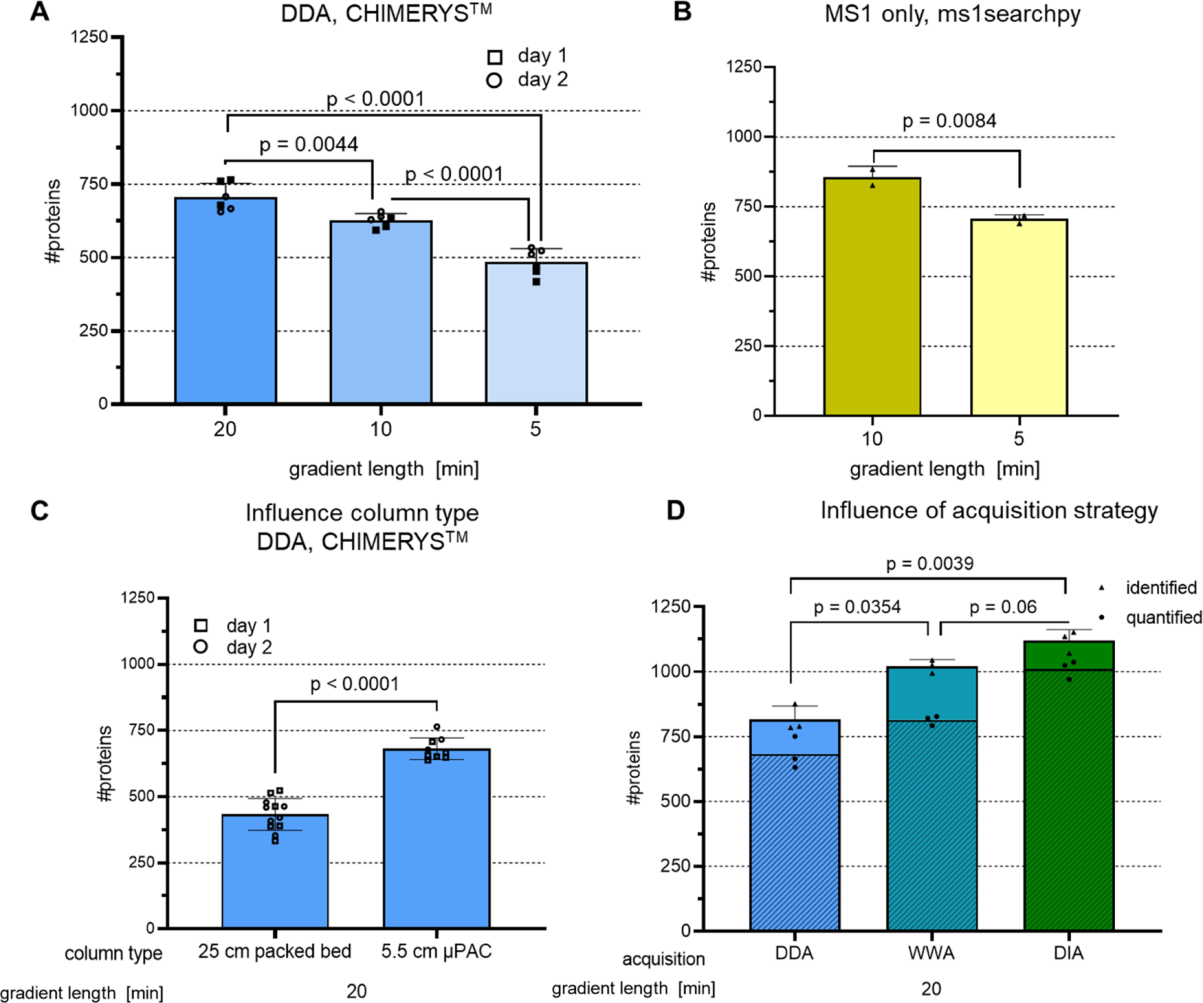
Influence of gradient length, column type, and data acquisition strategy on protein IDs of single-cell level inputs. 250 pg HeLa bulk digest was used for injection into LC-MS, each bar represents protein IDs on 1% FDR level, and error bars indicate standard deviations. To estimate significance, an unpaired two-tailed Student’s *t*-test was performed, and the resulting *p-*values are depicted above each compared data set. (A) Peptides were separated on a 5.5 cm μPAC column, data was acquired using DDA and analyzed using CHIMERYS, *n* = 6 technical replicates measured on 2 different days. (B) Same as A, but data analysis was performed using ms1searchpy,^25^*n* = 3 technical replicates. (C) Peptides were separated either on a 25 cm packed bed column or on a 5.5 cm μPAC column, and data was acquired using DDA and analyzed using CHIMERYS, *n* = 12 technical replicates measured on 2 different days. (D) Same as A, but using either DDA (2 *m*/*z* isolation width), WWA^22^ (12 *m*/*z* isolation width), or DIA (variable windows 8–25 *m*/*z* isolation width) as an acquisition strategy. DDA and WWA data were analyzed using CHIMERYS, DIA data was analyzed using Spectronaut in direct DIA mode, identified + quantified proteins are shown shaded, *n* = 3 technical replicates.

In parallel to parameter tuning for loss-free and robust single-cell sample preparation, we aimed to improve proteome coverage by optimizing data acquisition and analysis as well. In the first step, we compared two column types for their chromatographic performance: a classical packed bed column (CSH C18 Column, 130 Å, 1.7 μm, 75 μm × 250 mm, Waters) that was previously used in our lab and a μPAC column (brick shape pillars, 5.5 cm, prototype column, Thermo Fisher Scientific), which we recently successfully tested for low-input amounts as well as short gradients.^22^ Although the μPAC column is only 5.5 cm long, its internal flow path is roughly 50 cm, leading to a sufficient surface area, enabling powerful separation. The median FWHM peak width obtained using the packed bed column was 3.84 vs 4.77 s for the μPAC. The μPAC column clearly and reproducibly outperformed its conventional counterpart in ID numbers (Figure 4C), which is why we used it for the following optimizations as well. Despite slightly broadened peak widths, the resulting reporter ion intensity and, thus, average peak area is increased. This is likely reasoned by reduced on-column losses, especially of hydrophobic peptides and reduced on-column oxidation.^23,24^

Minimizing runtimes using short gradients offers the potential advantages of increased throughput and sensitivity due to sharper peak shapes. Unfortunately, reducing an already short 20 min active gradient to 5 min lowers protein IDs from 705 to 485 proteins or by about 30% (Figure 4A). The resulting peaks are sharp, with a median FWHM of 4.77 and 2.9 s for the 20 and 5 min gradients, respectively, which is a prerequisite for high sensitivity. However, both the 20 and the 5 min gradients make it difficult for a Thermo Scientific Orbitrap mass analyzer to record enough MS2 spectra to obtain the desired IDs. To address the need for increased throughput while achieving sufficient proteome coverage, the mass spectrometer duty cycle can be reduced by omitting requirements for fragment spectra. The first successful attempt to perform proteome-wide analyses using very short gradients of only 5 min was carried out by Ivanov and co-workers in 2020.^25,26^ Their approach was adopted for the single-cell workflow experiments described here. The 5.5 cm μPAC HPLC column was used, which due to its low backpressure (see Supporting Information Figure 7), allowed for fast loading and re-equilibration at 1 μL/min in a total runtime of 7.4 min. For short gradients, the MS1-only approach outperformed standard DDA. Roughly the same number of proteins were identified in the 5 and 20 min active gradients (Figure 4A,B). Of those proteins found in the 20 min DDA run, approximately 70% were identified in the 5 min MS1 run, which is comparable to published results for higher input samples^25^ (Supporting Information Figure 2).

Different mass spectrometer data acquisition strategies were then tested using the 20 min active gradient (Figure 4D). The proteome depth obtained using DDA was improved by widening the precursor isolation window from 2 to 12 *m*/*z*. Using such a WWA^22^ on purpose generated chimeric spectra and led to the identification of more than one peptide from a single spectrum when the CHIMERYS intelligent search algorithm was used. Variable DIA windows of up to 25 *m*/*z* for cofragmentation of ions significantly increased protein IDs to more than 1100 from 250 pg HeLa. The picture obtained for identification numbers was mirrored when looking at quantified proteins (depicted as a shaded area within the bar plot of Figure 4D) 83, 80, and 90% of all identified proteins were quantified for DDA, WWA, and DIA, respectively. The quality of quantification was however best for WWA with obtained median coefficients of variation (CV) of protein quantities of 17, 9.5, and 32% for DDA, WWA, and DIA, respectively (see also Supporting Information Figure 6 for single-cell stock samples).

**Figure 5:**
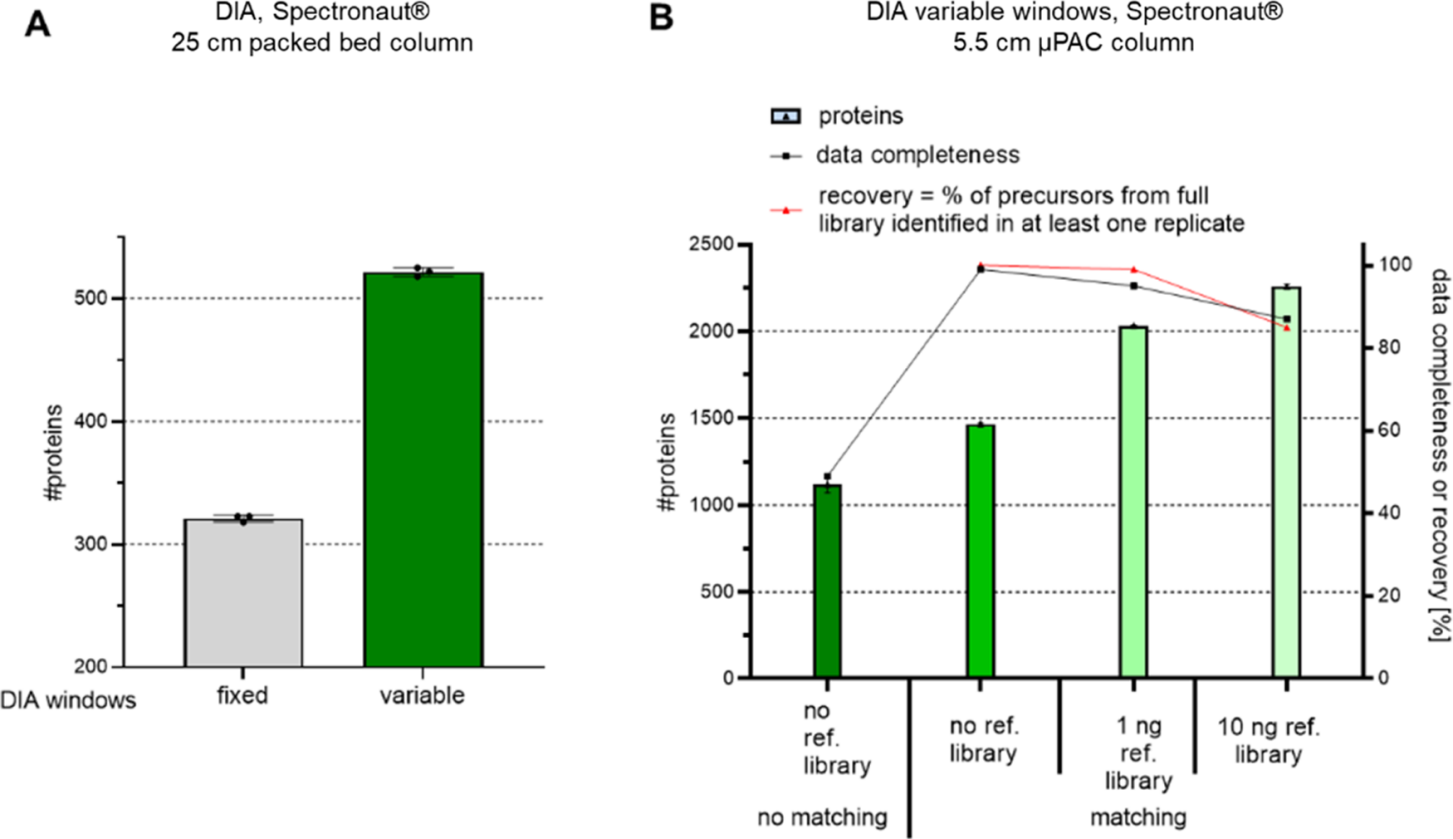
Benchmarking DIA methods and enrichment of searches to boost protein IDs of single-cell level inputs. 250 pg HeLa bulk digest was prepared for single-cell LC-MS analysis for each experiment; bars and error bars show the protein IDs at the 1% FDR level with their standard deviations. (A) Peptides were separated on a 25 cm packed bed column. Data were acquired using a fixed window size of 8 *m*/*z* or variable windows ranging from 8–25 *m*/*z* and then analyzed using Spectronaut, with *n* = 3 technical replicates. (B) Peptides were separated on a 5.5 cm μPAC HPLC column. Data were acquired using variable windows ranging from 8–25 *m*/*z* and then analyzed using Spectronaut either separately (no matching) or together (matching between runs), with or without enrichment using data from higher input experiments, with *n* = 3 technical replicates each.

Due to the superiority of the DIA, we investigated this strategy in more detail. Figure 5A shows the advantage of using variable rather than fixed windows. Using variable windows reduces the duty cycle because broader windows are used in *m*/*z* regions where few ions are expected. The mass spectrometer parameters used are provided in Supporting Information Table 2. Similar to the results obtained for the DDA experiments, protein IDs were increased using DIA, in this case by about a factor of two, when the μPAC HPLC column was used instead of the packed bed column (Figure 5A vs5B). In both experiments, the same method and gradient were used (dark green bar, 20 min gradient, variable windows). To dig deeper into the proteome and improve data completeness across replicates, matching between runs was performed (Figure 5B, no reference library). Across three replicates, data completeness increased to nearly 100% with almost 1500 proteins identified from 250 pg sample using a direct DIA search. Enriching the direct DIA search data with data obtained from higher input samples of 1 or 10 ng improved matching, boosting protein IDs to more than 2000 and 2200, respectively. Enlarging the library size, however, led to identifications not possible in all replicates (lower recovery), reducing data completeness to approximately 80% when the largest library was used. Of note, these results are in line with our observations made on a timsTOF with DIA-PASEF and using a library for enrichment using single cells (Pichler et al., manuscript in preparation).

**Figure 6:**
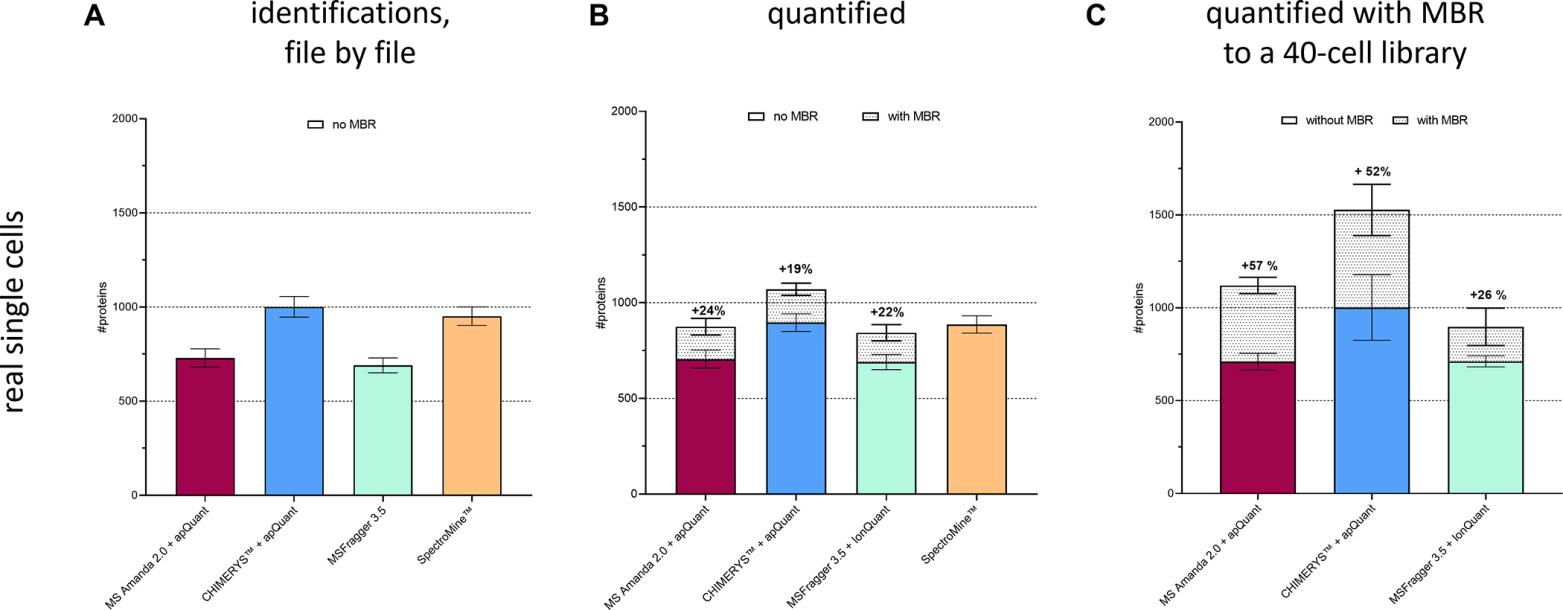
Benchmark of different data analysis tools and effect of enrichment for DDA runs. Individual HeLa cells were processed using the improved workflow. Peptides were separated using a 20 min active gradient, and data were acquired using a DDA method with a 2 *m*/*z* isolation width. (A) Data analysis was performed using each evaluated software tool at 1% FDR on the protein level, and raw files were analyzed file-by-file. Bars show the average number of identified proteins *n* = 5 replicates. (B) Same as A, but raw files were processed together to allow for MBR; bars show the number of quantified proteins. (C) Same as B, but analyzed together with five runs of 40× HeLa cells each to enrich matching.

Using match between runs (MBR) also improved the quantification results for DDA-based single-cell analysis. Five replicates of one individual HeLa cell each were analyzed by employing four of the commonly used data analysis algorithms (Figure 6A). The number of identified proteins of MS Amanda^27^ and MSFragger^29^ show similar results (mean values 730 and 690), as do the mean values of CHIMERYS and SpectroMine (1001 and 951). For quantification, MS Amanda and CHIMERYS algorithms were each evaluated in combination with apQuant^28^ within Proteome Discoverer (Thermo Scientific). MSFragger was used in combination with IonQuant^30^ (Figure 6B). For all, MS Amanda, CHIMERYS, and SpectroMine at ≥90% of all proteins were quantified. For MSFragger, it is only possible to display proteins that were identified and quantified. Using MBR yielded more than 24, 19, and 22% extra quantified proteins for MS Amanda, CHIMERYS, and MSFragger, respectively. As SpectroMine does not support MBR, it was excluded from this comparison. To improve sensitivity, five replicates of 40 HeLa cells were analyzed together with single-cell runs to provide more potential matches across files (Figure 6C). As a result, the matching library was increased to 7327 peptides and 1201 proteins for the MS Amanda searches and to 16,229 peptides and 2955 proteins for the CHIMERYS searches. The resulting protein quantification in single cells improved to 1120 and 1527 proteins on average for MS Amanda and CHIMERYS searches, respectively. In contrast, the result using MSFragger in combination with the additional 40 cell files was not significantly changed compared to using it without a library. With or without MBR, CHIMERYS outperformed all the other search engines tested, making it the preferred choice for DDA and WWA searches in this study. Of note, the use of our enrichment library did not negatively influence quantification accuracy as depicted in similar CV values (Supporting Information Figure 9).

### Proof of Principle Studies

#### Proteome Depth

**Figure 7:**
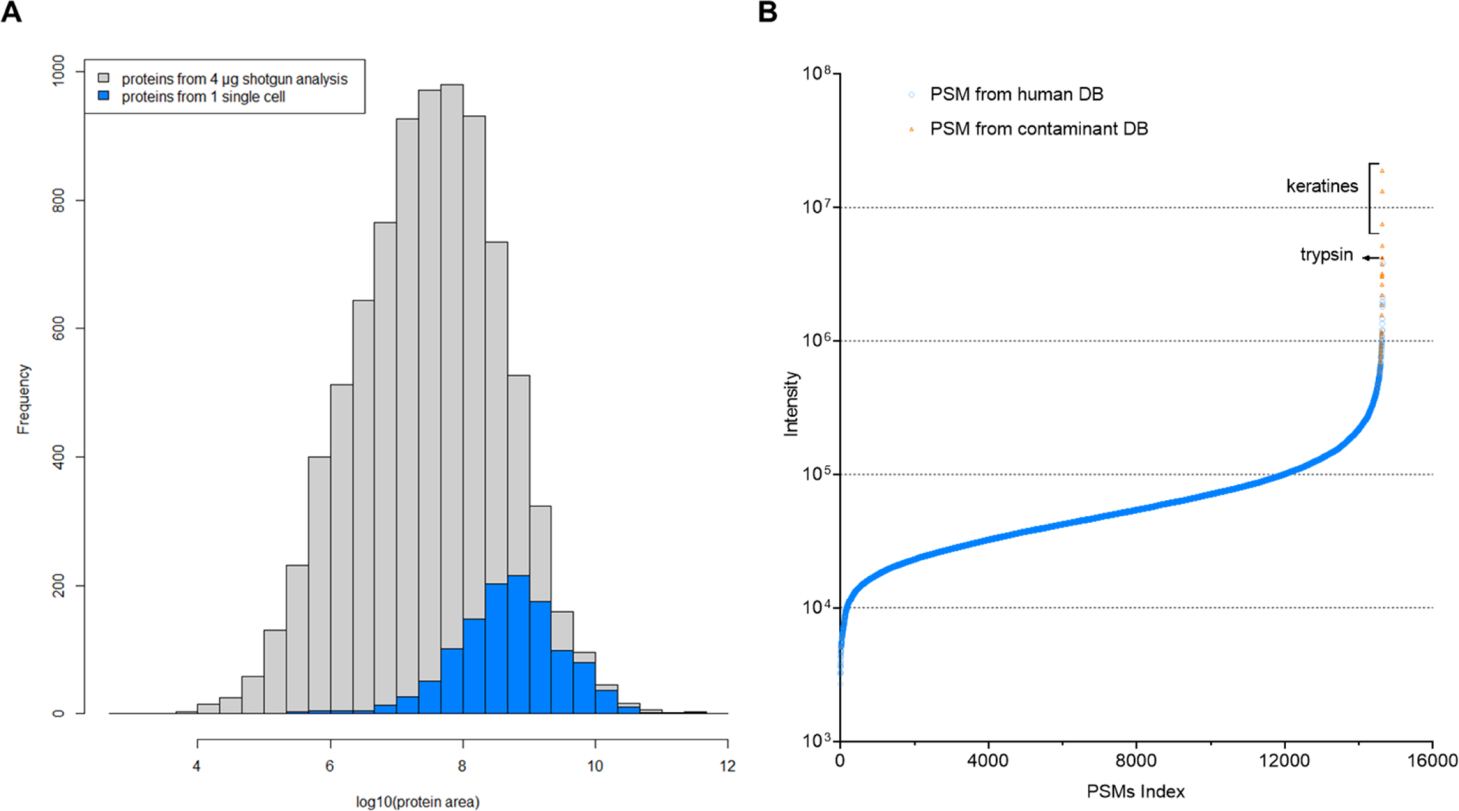
Proteome depth reached in single-cell experiments using DDA and label-free strategies. Data from a single HeLa cell obtained using the improved workflow, acquired in DDA mode and analyzed using CHIMERYS. (A) Proteins, excluding contaminants, identified from a single cell, plotted on the abundance distribution of all proteins identified in a 4 h shotgun run from 4 μg HeLa digest. LFQ was performed using apQuant. (B) PSMs identified in a single-cell analysis, indexed based on their precursor intensity. PSM matches originating from the contaminant database (DB) are highlighted in orange.

Reaching sufficient proteomic depth is essential for single-cell workflows that aim to investigate cellular heterogeneity. To estimate the sensitivity of the workflow presented here, all proteins identified from a single-cell run that yielded more than 1000 proteins were plotted into a histogram showing protein IDs obtained from a high-input sample with more than 8500 proteins quantified. As shown in Figure 7A, the single-cell analysis results predominantly covered proteins that are most abundant in the proteome. Nevertheless, the identified proteins ranged over more than four orders of magnitude in abundance, which is sufficient to allow investigation of differences in protein abundance. This broad dynamic range covered by the single-cell run was estimated by plotting all PSM intensities (Figure 7B). Notably, the most abundant hits were contaminants introduced during sample preparation, mostly keratins and trypsin, which are added in excess for digestion.

To ensure that those IDs that are of low precursor signal intensity and of a spectrum quality that is just accepted by the 1% FDR threshold, we performed an additional analysis, including an entrapment of the yest proteome into the database. We found that 1.5% of all peptides and 1.3% of all proteins in the result file originated from the yeast proteome, which indicates a proper overall FDR calculation. Furthermore, those PSMs identified from yeast are not accumulated in the low abundant fraction but are evenly distributed across the whole intensity range. This indicates that not only noise is reported in the low abundant fraction, but also the achieved dynamic range is truly in the range of four orders of magnitude (Supporting Information Figure 3).

#### Application of Optimal Workflow Parameters to Single-Cell Analysis of Two Human Cell Lines—Proof of Principle Study

**Figure 8:**
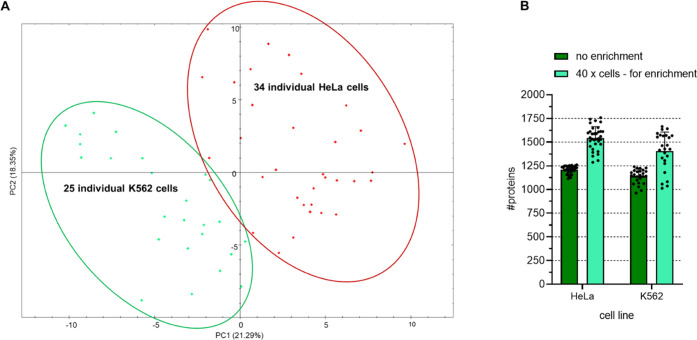
Application of optimal sample preparation, data acquisition, and data analysis parameters to single-cell analysis of two human cell lines. Individual HeLa or K562 cells were processed using the improved workflow, and the resulting peptides were separated using a 5.5 cm μPAC HPLC column in a 20 min active gradient. Data acquisition was by DIA with variable window sizes. Spectronaut was used for data analysis with 1% FDR at the protein level and MBR. (A) Principal component analysis in Spectronaut. Each dot represents one cell, with HeLa cells shown in red and K562 cells shown in green. (B) Bar chart comparison of the average number of identified proteins when analyzing data in the direct DIA mode without data enrichment, with data enrichment from 4 replicates, each originating from 40 HeLa or 40 K562 cells.

To evaluate our improved workflow, individual HeLa and K652 cells were isolated, processed, and analyzed using the best-found sample preparation conditions. Cells were kept hydrated, trypsin was added twice, and DMSO was supplemented for storage. Peptides were separated using a 20 min active gradient on a 5.5 cm μPAC HPLC column, and data were acquired using a DIA method with variable windows. On average, 1208 and 1139 proteins were identified from a single HeLa or K562 cell, respectively (Figure 8B). Matching to measurements of 40 HeLa or K562 cells boosted IDs to 1543 and 1404 proteins, respectively. Even with relatively few replicates, clear separation of cell types was obtained based on their proteome abundance variation (Figure 8A), indicating that the workflow is sensitive enough to enable investigation of cellular heterogeneity. This is mirrored when visualizing this data using a dendrogram (Supporting Information Figure 5), where we also included data from 40 cells measured (used for enrichment) or from a no-cell control sample. However, variability within each cell type is high as well. Our data suggests that this is reasoned by differences in age, size, or cell cycle phase of the used individual cells. We generated a sample under the exact same conditions but isolated 50× cells into one well. From that, we injected the volume corresponding to one individual cell repetitively. Using these “single-cell stocks,” we eliminate biological variability within each cell population but maintain all other single-cell workflow conditions. Using single-cell stocks and the same DIA method as used for real single cells, the resulting CV of peptide abundance was reduced from 61 to 25% (for HeLa), and PCA-based separation is much clearer (Supporting Information Figure 6).

## Conclusions

We here improved a sample preparation workflow for single-cell proteomics. The most important parameter to minimize losses of such ultralow input samples seems to perform all steps in a single pot. By omitting any transfer step but injecting directly from a 384-well plate, not only proteome coverage but also reproducibility was improved. The wells of a 384 plate thereby serve for all workflow steps from cell isolation to injection into the LC-MS system. Furthermore, preventing the drying of samples and the addition of fresh protease after 30 min clearly improved our results. We hypothesize that this not only elongates the total time of efficient digestion but also reduces adsorption of hydrophobic peptides on the well walls. This effect was supported by supplementation of DMSO for sample storage, keeping peptides solubilized.

The CellenONE robot enabled effective and semi-automated cell isolation, served as an incubation chamber with controlled temperature and humidity for lysis and digestion, and automatically performed hydration. Although this study was performed using the CellenONE instrument for all steps except cell isolation, the workflow could be easily implemented without it, since the volumes used are reasonably pipette-able (1 μL), and sample preparation is performed in standard 384-well plates. The 384-well plates are also compatible with alternative cell isolation machines, such as FACS devices, which are available in many labs.

Because a comprehensive workflow includes more than just sample preparation, the complete set of steps, including LC separation and MS data acquisition, analysis, and interpretation, were also evaluated and optimized. The short μPAC HPLC column, with its highly ordered and superficially porous brick-shaped pillars, provided excellent peptide separation and fast loading and equilibration due to exceedingly low backpressure. While LC-MS analysis in 5 min gradients is possible and generated more than 700 average protein IDs using MS1-only acquisition, best results were obtained using a 20 min active gradient with a total run-to-runtime of 39 min. With this cycle time, 37 samples can be analyzed per day. Though this is low throughput compared to multiplexed workflows, multiplexed workflows can suffer from ratio compression and precursor co-isolation, which interfere with accurate quantification.^14,31−33^ Because LFQ strategies offer unbiased and accurate quantification and are fully compatible with DIA, they are preferred when high throughput is not required.

The improved workflow identified more than 1000 proteins over four orders of magnitude in abundance from a single cell using DDA and data processing without MBR. More than 1500 proteins were quantified when data were processed with MBR. Proteomic depth was further improved using WWA or DIA instead of DDA, providing identification of up to 2250 proteins in a single cell and enabling differentiation of cell types based on protein abundance differences. Therefore, the use of a 40× cell library was shown to be advantageous to further improve proteome coverage for single-cell samples. When comparing the different software options, although CHIMERYS was able to achieve the highest number of proteins identified, for quantification, the lowest CV was achieved with analysis using MSFragger in combination with IonQuant and FDR-controlled MBR. Of note, DIA outperformed WWA in terms of ID numbers in our hands, but WWA showed improved CV for quantified peptides (Supporting Information Figure 6). In contrast to our results, the Kelly lab^34^ reported WWA outperforming DIA in a single-cell context using a different LC-MS setup and DIA method. This suggests that both methods might be the first choice for a single-cell study, depending on the LC-MS setup used, acquisition method details, as well as on the question of whether maximizing IDs or quantitative accuracy is more relevant to a project.

Easy to implement, robust, and sensitive: the improved workflow has the potential to become the gold standard for studies aiming to dig as deeply as possible into the proteome of individual cells without the need to use carriers or labeling. In addition, the here developed techniques are not only suitable for single-cell applications but also in general for all proteomic studies that are limited in input amount requiring loss-free handling and will therefore be highly valuable for a broad scientific community.

## Data Availability

All raw and result files are available for download at MassIVE using the identifier MSV000091156.

## Supporting Information Available

Materials and methods: label-free MS/MS method for DDA, DIA, and MS1 only (Table S1); summary of the variable isolation windows used for DIA (Table S2); summary of data analysis settings for different software and search algorithms (Table S3); single-cell isolation by visual detection using the cellenONE (Figure S1); Venn diagram showing proteins commonly found in a representative replicate from 250 pg HeLa (Figure S2); reachable dynamic range from one cell, label-free and using DDA (Figure S3); improving recovery and reproducibility by tuning workflow parameters (Figure S4); dendrogram showing differentiation of cell types from 40× cell input and no cell controls (Figure S5); analysis of two human cell lines using single-cell stocks and WWA or DIA (Figure S6); representative pressure curves (Figure S7); effect of manual pipetting for master mix dispensing and hydration (Figure S8); and analysis of five real single HeLa cells with MBR using a 40× HeLa library for enrichment (Figure S9) (PDF)
