# Supplementary material for: Robust and Easy-to-Use One-Pot Workflow for Label-Free Single-Cell Proteomics
Source: Anal Chem. 2023 Feb 20;95(9):4435–45. doi: 10.1021/acs.analchem.2c05022 (PMC9996606; doi:10.1021/acs.analchem.2c05022)
Supplement: Supplementary file 1 — ac2c05022_si_001.pdf [file ac2c05022_si_001.pdf]

Supporting Information

Robust and easy-to-use one pot workflow for label free single cell proteomics

Manuel Matzinger[1, #, *], Elisabeth Müller[1, #], Gerhard Dürnberger[3], Peter Pichler[1], Karl Mechtler[1,2,3,*]

[1]Institute of Molecular Pathology (IMP), Campus-Vienna-Biocenter 1, 1030, Vienna, Austria

[2]Institute of Molecular Biotechnology of the Austrian Academy of Sciences, Dr. Bohrgasse 3, 1030, Vienna, Austria

[3]Gregor Mendel Institute of Molecular Plant Biology (GMI) of the Austrian Academy of Sciences, Dr. Bohrgasse 3, 1030, Vienna, Austria.

# MM and EM contributed equally

*Correspondence to: manuel.matzinger@imp.ac.at, karl.mechtler@imp.ac.at

Table of content

Methods: Sample preparation, LC-MS and data analysis

Supplemental Table 1: Label free MS/MS method for DDA, DIA and MS1only

Supplemental Table 2: Summary of the variable isolation windows used for DIA

Supplemental Table 3: Summary of data analysis settings for different software and search algorithms

Supplemental Figure 1: Single cell isolation by visual detection using the cellenONE®.

Supplemental Figure 2: Venn diagram showing proteins commonly found in a representative replicate from 250 pg HeLa

Supplemental Figure 3: Reachable dynamic range from one cell, label free and using DDA

Supplemental Figure 4: Improving recovery and reproducibility by tuning workflow parameters

Supplemental Figure 5: Dendrogram showing differentiation of cell types from 40 x cell input and no cell controls

Supplemental Figure 6: Analysis of two human cell lines using single cell stocks and WWA or DIA

Supplemental Figure 7: Representative pressure curves

Supplemental Figure 8: Effect of manual pipetting for mastermix dispensing and hydration

Supplemental Figure 9: Analysis of five real single HeLa cells with MBR using a 40x HeLa library for enrichment

References

METHODS

Sample preparation

HeLa cells were cultured at 37˚C and 5% CO_2 in Dulbecco's Modified Eagle's Medium (DMEM) supplemented with 10% FBS (10270, Fisher Scientific™, USA), 1x penicillin-streptomycin (P0781-100ML, Sigma Aldrich, Israel) and 100X L-Glut 200 mM (250030-024, Thermo Scientific™, Germany). After trypsinization with 0.05% Trypsin-EDTA (25300-054, Thermo Scientific™, USA) cells are washed 3x with phosphate-buffered-saline (PBS). K562- WT cells were cultured at 37˚C and 5% CO_2 in Gibco Roswell Park Memorial Institute (RPMI -1640) medium (Fisher Scientific™, USA). For harves K562 were pelleted by centrifugation (117g, 4˚C) and washed 3x with phosphate-buffered-saline (PBS). All cells were resuspended in PBS at 200 cells/µL for isolation within the CellenONE®.

Benchmarking experiments mimicking single cell digests were prepared from dilutions of the Pierce™ HeLa (#1862824, Thermo Scientific™) protein digest standard in 0.1% TFA with or without 5 % DMSO supplemented as indicated.

Sample lysis and digestion is performed within a clean 384 well plate (Thermo Scientific™ Armadillo PCR Plate, 384-well, #_12657516) inside the CellenONE®. 1 µL of a master mix containing 0.2% DDM (D4641-500MG, Sigma Aldrich, Germany), 100mM TEAB (17902-500ML, Fluka Analytical, Switzerland), 3 ng/µL trypsin (Trypsin Gold, V5280, Promega, USA), 0.01 % enhancer (ProteaseMAX™, V2071, Promega, USA) is predispensed into the wells. For comparison of trypsin types, the following proteases have been used: Trypsin Gold, V5280, Promega, USA, batch A: LOT:0000478374, batch B: LOT: 0000500298 and Trypsin Platinum, VA9000, Promega, USA, batch A: LOT: 0000478061, batch B: LOT:0000545454.

To limit evaporation humidity is set to the 85%. Individual cells at 18 - 25 µm diameter and a max elongation of 1.5 are sorted by the CellenONE® into the respective wells. This is followed by incubation for 2 h at 50 ˚C at 85% relative humidity inside the instrument. Samples were kept hydrated every 15 min by automated addition of 500 nL water to each well. After 30 min of incubation, additional 500nL of 3ng/µL trypsin are added which replaces one hydration step. After lysis and digestion, 3.5 µL of 0.1% TFA with 5% DMSO were added to the respective wells for quenching and storage. Samples were either directly injected from the 384 well plate or transferred to a PEG coated PCR tubes (PCR-02-L-C, Axygen®) or to the autosampler glass vials (601801655, Thermo Scientific).

LC-MS/MS analysis

Samples were analyzed using the Ultimate 3000 RLS-nano high-performance liquid chromatography (Thermo Scientific™) or the Vanquish™ Neo UHPLC system (VN-S10-A01, Thermo Scientific™). Peptides were separated on a 5.5 cm brick shape pillar column prototype (µPACTM, Thermo Fisher) or on a nanoEase M/Z Peptide CSH C18 column (130Å, 1.7 µm, 75 µm x250 mm, 18600810, Waters, Germany) as indicated in a trap and elute setting using an Acclaim™ PepMap™ 100 C18 trapping column (5 µM, 0.3 mm x 5 mm, Thermo Scientific™). To ensure complete loading of single cell samples (filled up with 3.5 µL 0.1% TFA/5% DMSO) an injection volume of 4 µL was chosen, with the injection needle set to 100 µm above the well bottoms during injection. Visual inspection after injection suggested no, or close to no residual (< 1%) volume left inside the wells after injection. Analytes were eluted onto the analytical column by backflushing the trapping column. All columns were operated at 50˚C and connected to an EASY-Spray™ bullet emitter (10 µm ID, ES993; Thermo Fisher Scientific) An electrospray voltage of 2.4 kV was applied at the integrated liquid junction of EASY-Spray™ emitter. Mass spectrometry measurement was performed by an Orbitrap Exploris™ 480 Mass Spectrometer (Thermo Scientific™) equipped with a FAIMS electrode (Thermo Scientific™). A single CV of -50 was used as this was shown to improve sensitivity and proteome coverage for low load samples and short gradients.[1,2]

If not otherwise noted, peptide separation was performed at a constant flow rate of 250 nL/min using the following 20-min active gradient: 1% buffer B (acetonitrile (ACN) with 0.08% formic acid) and 99% buffer A (0.1% formic acid (FA)) from minute 0 to 2.5, rising to 2.5% B from minute 2.5 to 2.75, continuing to rise to 25% until minute 17, followed by an increase from 25 to 40% in the last 5 min. The column was washed by increasing buffer B to 97.5% until minute 22 for 10 min before buffer B

concentration was decreased back to 1% for re-equilibration. The gradient was shortened to 10 and 5 min for the benchmarking studies. When using the packed-bed column, re-equilibration was performed for 18 min at 250 nL/min. The µPAC™ HPLC column was used on the Vanquish Neo HPLC System with fast loading and equilibration at a maximum pressure of 350 bar and a maximum flowrate of 1 µL/min. For MS1-only acquisition, a linear gradient was applied that ranged from 1 to 40% buffer B only but with no other changes compared to the above-described gradient.

The mass spectrometer and FAIMS Pro Interface parameters are provided in Supplemental Table 1 and Supplemental Table 2.

Data analysis

If not otherwise noted, data analysis was performed using CHIMERYS™ as a Proteome Discoverer software node (v3.00.757, Thermo Scientific). Data were searched against the human reference database from Uniprot (version: 20.08.2021 UP000005640, 20300 sequences, 11359367 residues) and a customized contaminants database (364 sequences; 142459 residues). For evaluation of the FDR a yeast database was used (version 22.12.2022 UP000002311, 6050 sequences). Trypsin was specified as proteolytic enzyme, cleaving after lysine (K) and arginine (R) except when followed by proline (P) and up to two missed cleavages were allowed. Fragment mass tolerance was limited to 20 ppm and carbamidomethylation of cysteine (C) was set as a fixed modification and oxidation of methionine (M) as a variable modification. Identified spectra were rescored using Percolator[3] and results were filtered for 1% FDR on peptide and protein level. Abundance of identified peptides was determined by LFQ using IMP-apQuant[4].

For benchmarking purposes, data were also analyzed using MSAmanda (v2.0.0.19742)[5], SpectroMine™ (Version 3.2.220222.52329, Biognosys AG), MSFragger[6], and IonQuant[7] in FragPipe (Version 18.) For DIA data processing, Spectronaut™ (Version 16.1.220730.53000, Biognosys AG) was used in the directDIA mode. LFQ was performed with the build in MaxLFQ[8,9] using factory settings. For MS1 only, raw files were converted to mzML files using MSConvert[10] (v 3.0.21084) enabling the option Peak Picking. The resulting files were analyzed using ms1searchpy[11,12]. Detailed search settings can be found in Supplemental Table 3.

For MBR standard settings of each benchmarked software were used if not mentioned different. For apQuant (used in combination with Amanda and CHIMERYS) retention time tolerance was set to 0.5 min

and mass tolerance to 20 ppm. To cope with ultra-low input samples the minimal area for quantification was reduced to 5000. FDR was computed based on target and decoy extracted ion chromatograms (XICs), whereas decoy XICs are created by a constant shift in retention time and mass. The functionality XGBoost was used to optimally separate targets from decoys in a multidimensional; space of additional apQuant score components[4] and target XICs were filtered to obtain a final FDR of 1% at the XIC level. For IonQuant (used in MSFragger), retention time tolerance was set to 0.4 min and mass tolerance to 10 ppm, and FDR for MBR is set to 1 % and controlled in a target decoy manner as well.[7]

For DIA runs were searched with Spectronaut in directDIA mode using factory settings but removing Carbamidomethylation as modification for single cell samples. The search consists of two steps. In step one all raw files are searched for identification to generate the search space for the second cumulative search. All the identifications are controlled by FDR on the level of the generated library. FDR filters are applied on PSM, peptide and finally protein group level at 1%.

PCA plots were generated using the respective post-analysis function within Spectronaut. The dendrogram was generated using hierarchical clustering (R base function hclust). Pairwise sample distance is based on the correlation of log transformed abundances subtracted from one (d = 1-corr(log(a))) with d = distance, a = abundance matrix.

Supplementary Tables

Settings	DDA	DIA	MS1only
**Full Scan**			
Resolution MS1	*120000*	120000	120000
Scan range	*375-1200*	375-1200	375-1200
FAIMS CV	*-50*	-50	-40, -50, -60
AGC Target (%)	*300*	300	300
Maximum Injection time (s)	*Auto*	Auto	Auto
**MS2 scan**			
Number of dependent scans	*10*		
Minimum precursor intensity	$5.0*e^3$		
Charge state	*2-5*		
Dynamic exclusion time (s)	*120*		

Isolation window (m/z)	*2*		
HCD Collision Energy (%)	*30*	30	
Resolution MS2	*60000*	60000	
First mass (m/z)	*120*		
AGC Target (%)	*75*	75	
Maximum Injection Time (s)	*118*	118	

Supplemental Table 1: Label free MS/MS method for DDA, DIA and MS1only

Precursor mass range (m/z)	Isolation window (m/z)	Number of scan events
375-500	25	4
500-600	13	7
600-850	8	31
850-900	13	3
900-1200	25	9

Supplemental Table 2: Summary of the variable isolation windows used for DIA

	MSAmanda	SpectroMine®	Spectronaut™	FragPipe	Ms1searchpy
Proteolytic enzyme	Trypsin, cleavage at K and R if not followed by P				
Missed cleavages	max. 2				
Precursor mass tolerance	±5 ppm				± 8 ppm
Fragment tolerance	10 ppm				-
Minimum peptide length	6				7
Variable modifications	Oxidation at methionine, acetylation at protein N-terminus				Not defined
FDR (peptide, protein level)	1%				

Supplemental Table 3: Summary of data analysis settings for different software and search algorithms

Supplementary Figures

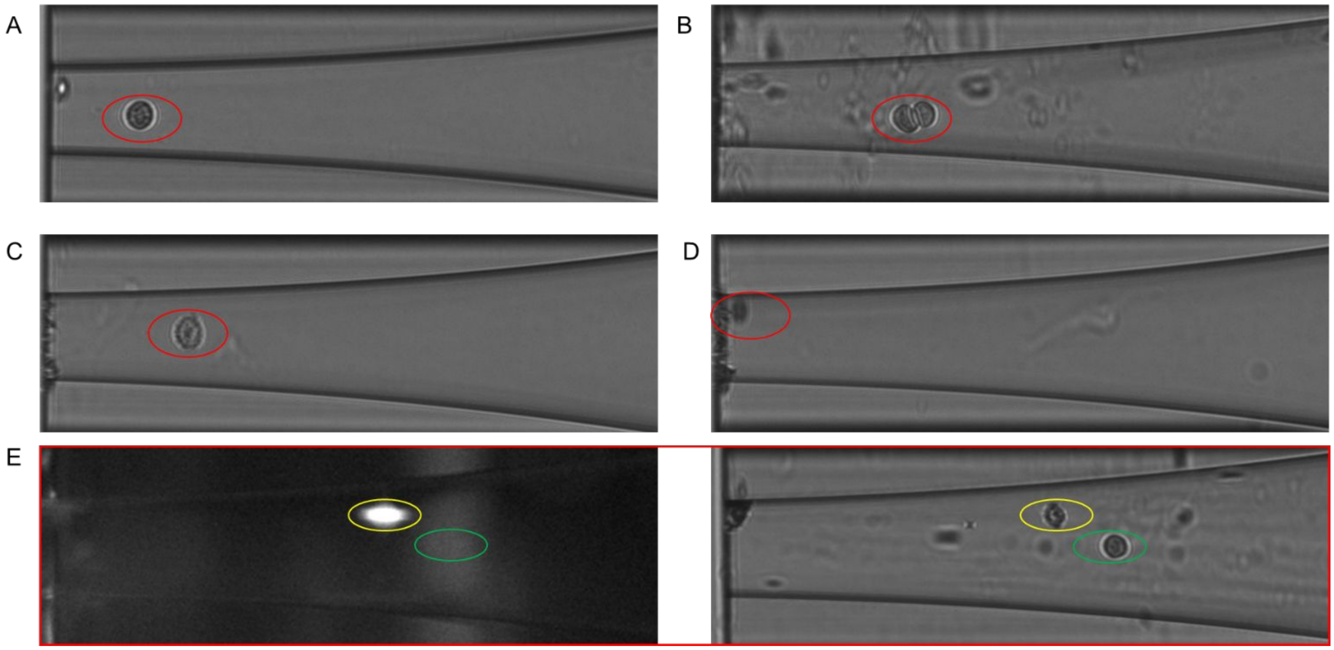

Supplemental Figure 1: ***Single cell isolation by visual detection using the cellenONE®*** *(**A**): One intact single HeLa cell is isolated. (**B**): Two cells are co-isolated by accident. (**C**): A single cell whose membrane looks very uneven and that might be inviable. (**D**): No cell is isolated by accident as a condensation droplet on the outer wall mimics a cell. (**E**) Validation of viability using HOECHS 33342 to stain condensed DNA of apoptotic cells, left: fluorescence image, right: brightfield image, apoptotic cell marked in yellow, healthy cell in green*

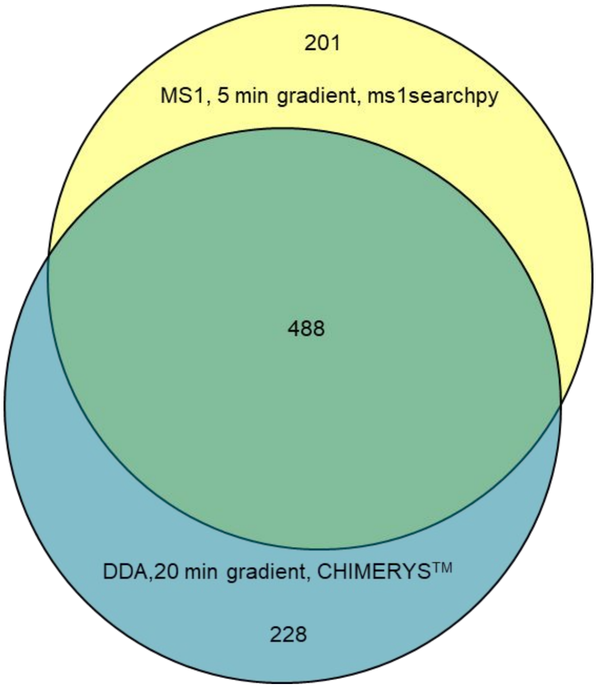

Supplemental Figure 2: ***Venn diagram showing proteins commonly found in a representative replicate from 250 pg HeLa.*** *A comparison of the proteins commonly found in a 5-min active gradient with MS1-level spectra used for identification (ms1serchpy) and in a 20-min active gradient, using DDA, and CHIMERYS™ for data analysis.*

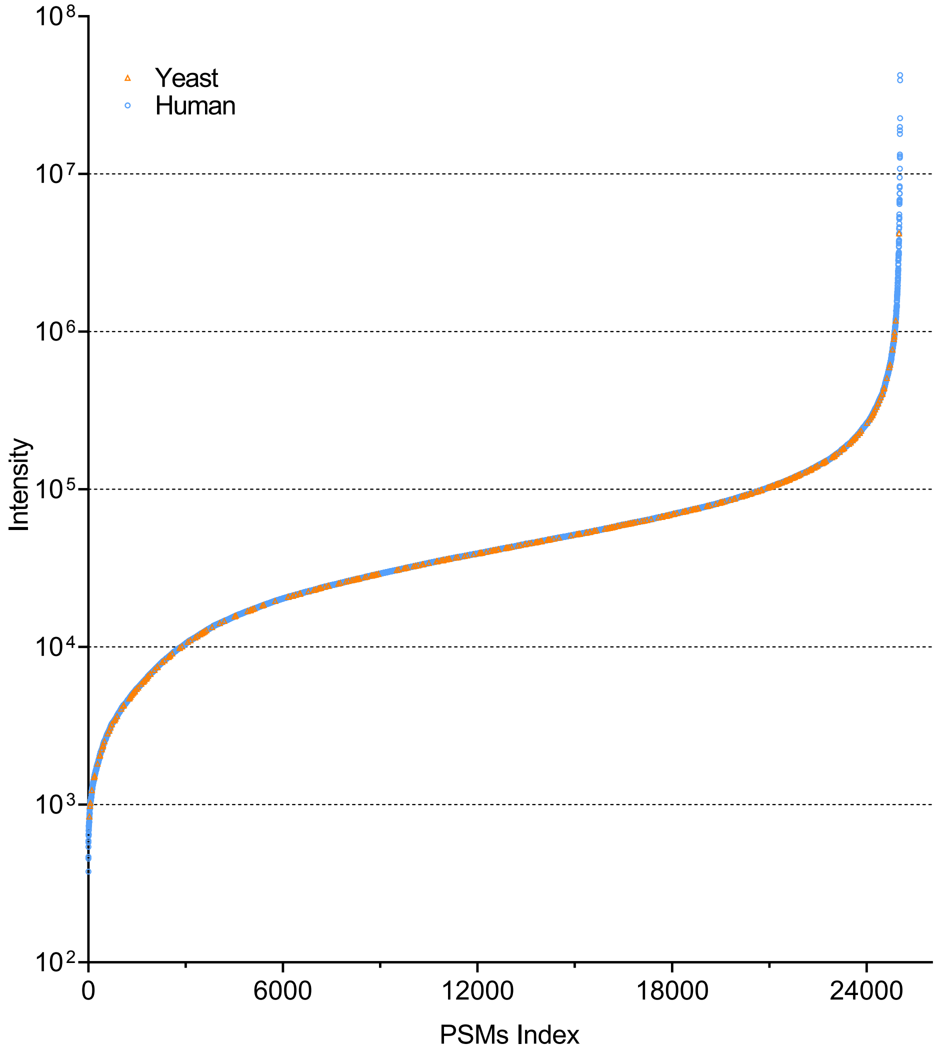

Supplemental Figure 3: ***Reachable dynamic range from one cell, label free and using DDA****. Data obtained from a single HeLa cell using the optimized workflow, acquired using a DDA method, and analyzed using CHIMERYS™. PSMs identified from the single-cell run are indexed based on their precursor intensity. PSM matches originating from human (24,654; 98.51%) are marked in blue, PSM matches from the yeast proteome database (374; 1.49%) are highlighted in orange.*

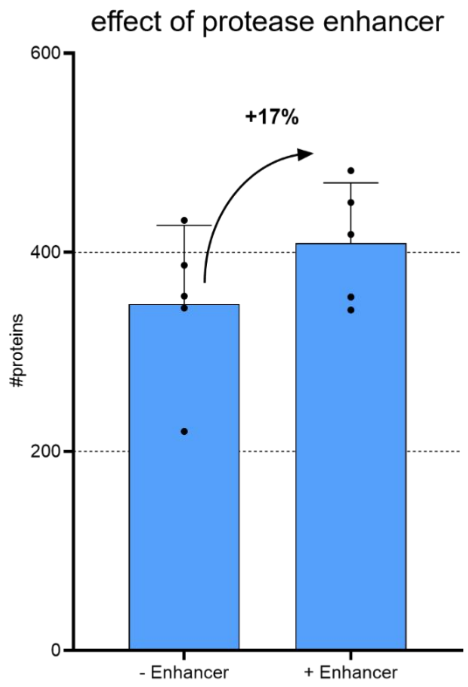

Supplemental Figure 4: ***Improving recovery and reproducibility by tuning workflow parameters****. Cells were digested with Trypsin Gold with and without the addition of the protease enhancer ProteaseMAX (Promega)*

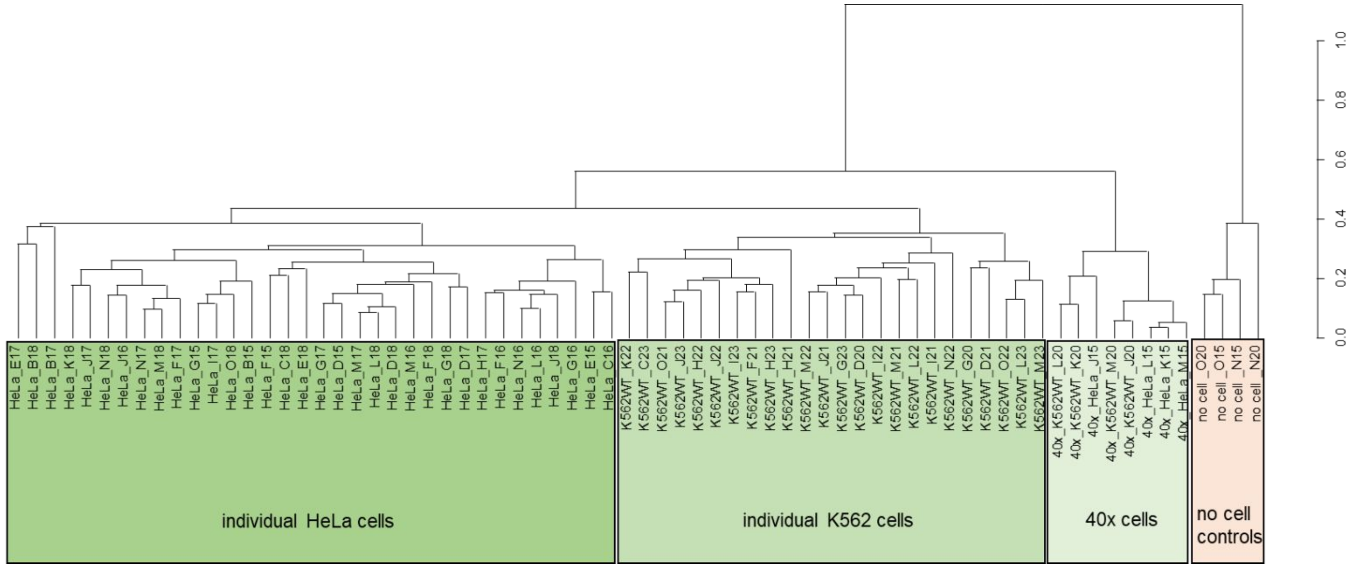

Supplemental Figure 5: ***Dendrogram showing differentiation of cell types from 40 x cell input and no cell controls****. Data from Figure 8 but including higher input (40 cells) and no input (no cell) data. No cell controls were processed within the cellenONE in the exact same way as all samples, but no cell was added.*

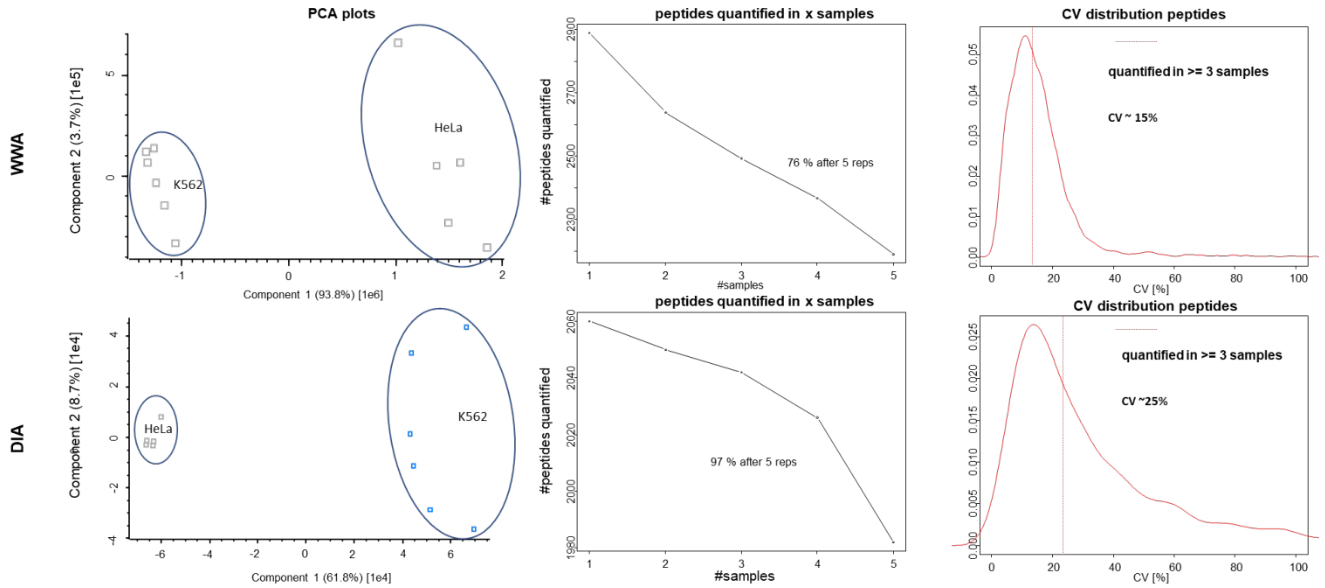

Supplemental Figure 6: ***Analysis of two human cell lines using single cell stocks and WWA or DIA****. 40x HeLa or K562 cells were prepared using the improved single cell protocol into one well. 1/40 of the total volume was injected for each replicate with n=5 and 6 technical replicates for HeLa and K562 respectively. Top: Acquired using WWA (iso = 12 Th), bottom: Acquired using DIA with variable windows (Supplemental Table 2). Left: PCA separation without any imputation. Middle: Number of peptides quantified in the HeLa replicates indicating the degree of data completeness after n=5, right: Distribution of CV of peptides quantified in at least 3 samples and median CV highlighted.*

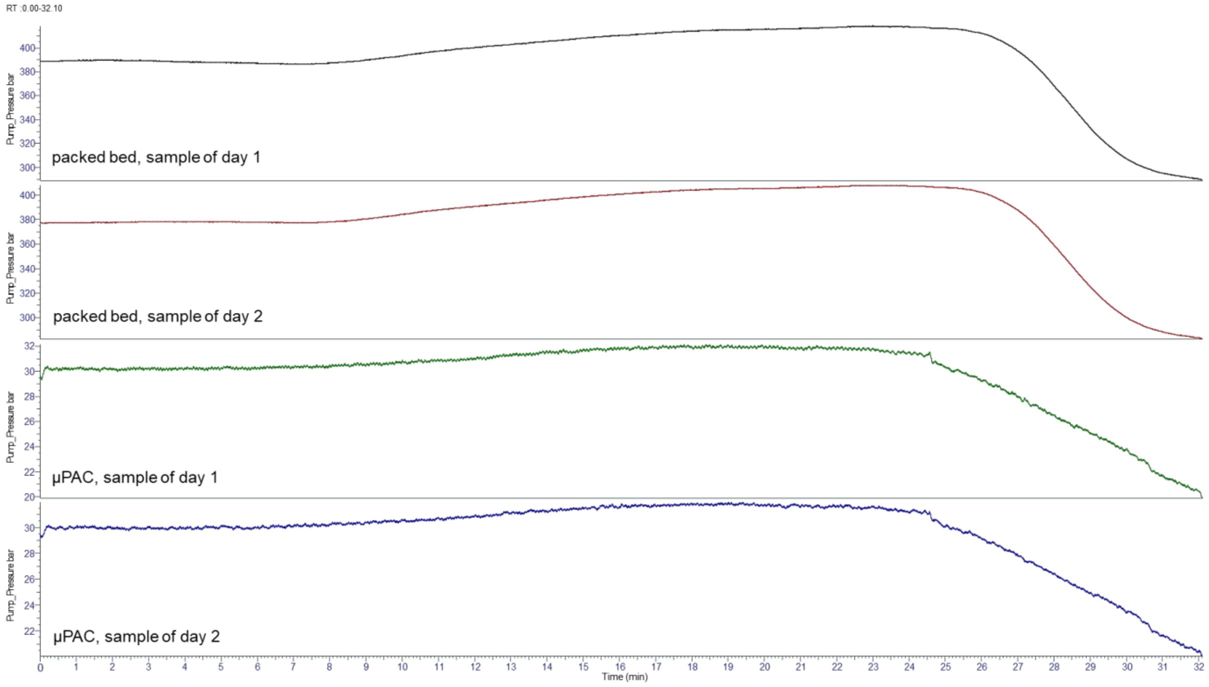

Supplemental Figure 7: ***Representative pressure curves:*** *Packed bed (CSH C18 Column, 130Å, 1.7 µm, 75 µm X 250 mm, Waters) and µPAC (5.5 cm prototype) columns. Example data of figure 4C within the main manuscript. 250 pg HeLa digest injected using a 20 min active gradient. All data on the recorded on the same system but with the analytical column exchanged.*

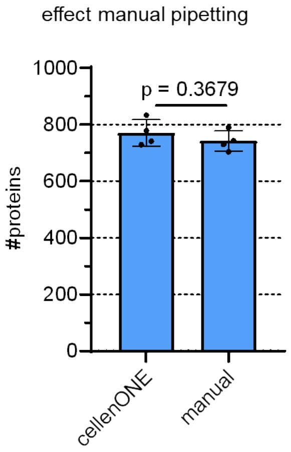

Supplemental Figure 8: ***Effect of manual pipetting for mastermix dispensing and hydration****. Protein IDs obtained from individual HeLa cells with the following workflow alteration: Mastermix was either dispensed to wells of the 384 well plate using the cellenONE or a multichannel pipette. Individual HeLa cells were isolated and incubated to 50°C using the cellenONE. Hydration with water was done either manually using a multichannel pipette followed by a quick spinning in the centrifuge or*

automated using the cellenONE. The bars and error bars show the protein IDs obtained with their standard deviations. An unpaired two tailed Students t-test was performed and resulting p values are depicted above, n =4 replicates.

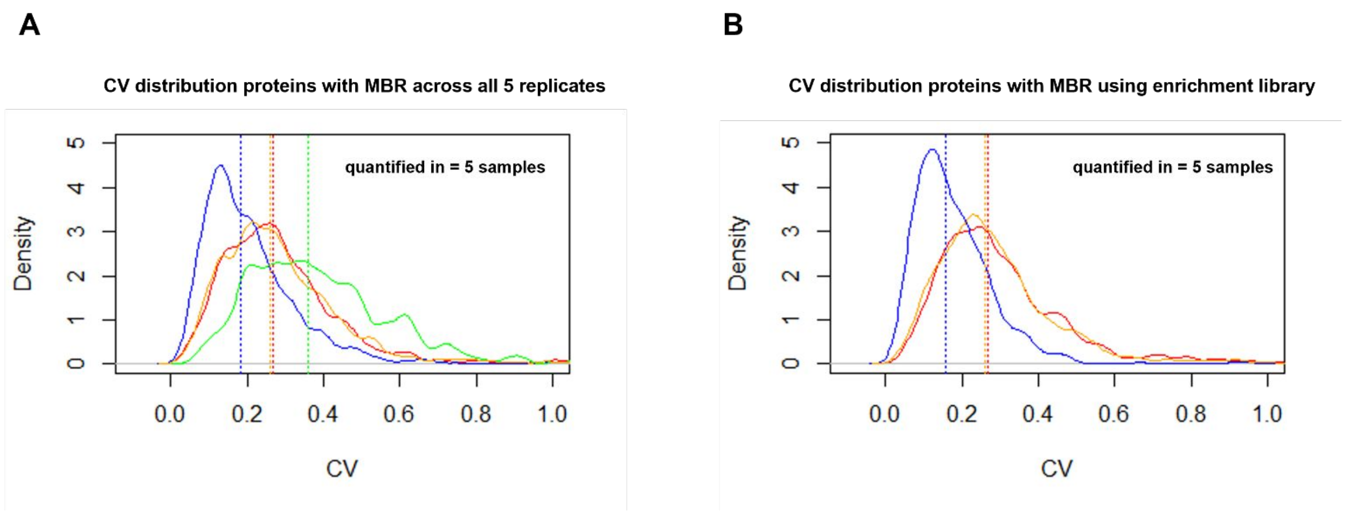

Supplemental Figure 9 ***Analysis of five real single HeLa cells with MBR using a 40x HeLa library for enrichment.*** *Distribution of CV of proteins quantified in all 5 samples and median CV highlighted. Green line shows data analyzed with SpectroMine™; Red line shows data analyzed with CHIMERYS™; Blue line shows data analyzed with MS Fragger; Orange line shows data analyzed with MS Amanda.* ***A****: Distribution of CV of proteins of 5 samples analyzed together are displayed. For all three software algorithms except SpectroMine™ MBR across all 5 replicates was performed.* ***B****: Distribution of CV of proteins of 5 samples analyzed together are displayed. MBR across the 5 replicates and the 40x cell library was performed.*

References

(1) Hebert, A. S.; Prasad, S.; Belford, M. W.; Bailey, D. J.; McAlister, G. C.; Abbatiello, S. E.; Huguet, R.; Wouters, E. R.; Dunyach, J.-J.; Brademan, D. R.; Westphall, M. S.; Coon, J. J. Comprehensive Single-Shot Proteomics with FAIMS on a Hybrid Orbitrap Mass Spectrometer. *Anal. Chem.* **2018**, *90* (15), 9529–9537. https://doi.org/10.1021/acs.analchem.8b02233.

(2) Stejskal, K.; Op de Beeck, J.; Dürnberger, G.; Jacobs, P.; Mechtler, K. Ultrasensitive NanoLC-MS of Subnanogram Protein Samples Using Second Generation Micropillar Array LC Technology with Orbitrap Exploris 480 and FAIMS PRO. *Anal. Chem.* **2021**, *93* (25), 8704–8710. https://doi.org/10.1021/acs.analchem.1c00990.

(3) The, M.; MacCoss, M. J.; Noble, W. S.; Käll, L. Fast and Accurate Protein False Discovery Rates on Large-Scale Proteomics Data Sets with Percolator 3.0. *J. Am. Soc. Mass Spectrom.* **2016**, *27* (11), 1719–1727. https://doi.org/10.1007/s13361-016-1460-7.

(4) Doblmann, J.; Dusberger, F.; Imre, R.; Hudecz, O.; Stanek, F.; Mechtler, K.; Dürnberger, G. ApQuant: Accurate Label-Free Quantification by Quality Filtering. *J. Proteome Res.* **2019**, *18* (1), 535–541. https://doi.org/10.1021/acs.jproteome.8b00113.

(5) Dorfer, V.; Pichler, P.; Stranzl, T.; Stadlmann, J.; Taus, T.; Winkler, S.; Mechtler, K. MS Amanda, a Universal Identification Algorithm Optimized for High Accuracy Tandem Mass Spectra. *J. Proteome Res.* **2014**, *13* (8), 3679–3684. https://doi.org/10.1021/pr500202e.

(6) Kong, A. T.; Leprevost, F. V.; Avtonomov, D. M.; Mellacheruvu, D.; Nesvizhskii, A. I. MSFragger: Ultrafast and Comprehensive Peptide Identification in Mass Spectrometry–Based Proteomics. *Nat. Methods* **2017**, *14* (5), 513–520. https://doi.org/10.1038/nmeth.4256.

(7) Yu, F.; Haynes, S. E.; Nesvizhskii, A. I. IonQuant Enables Accurate and Sensitive Label-Free Quantification With FDR-Controlled Match-Between-Runs. *Mol. Cell. Proteomics* **2021**, *20*, 100077. https://doi.org/10.1016/j.mcpro.2021.100077.

(8) Cox, J.; Hein, M. Y.; Luber, C. A.; Paron, I.; Nagaraj, N.; Mann, M. Accurate Proteome-Wide Label-Free Quantification by Delayed Normalization and Maximal Peptide Ratio Extraction, Termed MaxLFQ*. *Mol. Cell. Proteomics* **2014**, *13* (9), 2513–2526. https://doi.org/10.1074/mcp.M113.031591.

(9) Pham, T. V.; Henneman, A. A.; Jimenez, C. R. Iq: An R Package to Estimate Relative Protein Abundances from Ion Quantification in DIA-MS-Based Proteomics. *Bioinformatics* **2020**, *36* (8), 2611–2613. https://doi.org/10.1093/bioinformatics/btz961.

(10) Chambers, M. C.; Maclean, B.; Burke, R.; Amodei, D.; Ruderman, D. L.; Neumann, S.; Gatto, L.; Fischer, B.; Pratt, B.; Egertson, J.; Hoff, K.; Kessner, D.; Tasman, N.; Shulman, N.; Frewen, B.;

Baker, T. A.; Brusniak, M.-Y.; Paulse, C.; Creasy, D.; Flashner, L.; Kani, K.; Moulding, C.; Seymour, S. L.; Nuwaysir, L. M.; Lefebvre, B.; Kuhlmann, F.; Roark, J.; Rainer, P.; Detlev, S.; Hemenway, T.; Huhmer, A.; Langridge, J.; Connolly, B.; Chadick, T.; Holly, K.; Eckels, J.; Deutsch, E. W.; Moritz, R. L.; Katz, J. E.; Agus, D. B.; MacCoss, M.; Tabb, D. L.; Mallick, P. A Cross-Platform Toolkit for Mass Spectrometry and Proteomics. *Nat. Biotechnol.* **2012**, *30* (10), 918–920. https://doi.org/10.1038/nbt.2377.

(11) Ivanov, M. V.; Bubis, J. A.; Gorshkov, V.; Tarasova, I. A.; Levitsky, L. I.; Lobas, A. A.; Solovyeva, E. M.; Pridatchenko, M. L.; Kjeldsen, F.; Gorshkov, M. V. DirectMS1: MS/MS-Free Identification of 1000 Proteins of Cellular Proteomes in 5 Minutes. *Anal. Chem.* **2020**, *92* (6), 4326–4333. https://doi.org/10.1021/acs.analchem.9b05095.

(12) Ivanov, M. V.; Bubis, J. A.; Gorshkov, V.; Abdrakhimov, D. A.; Kjeldsen, F.; Gorshkov, M. V. Boosting MS1-Only Proteomics with Machine Learning Allows 2000 Protein Identifications in Single-Shot Human Proteome Analysis Using 5 Min HPLC Gradient. *J. Proteome Res.* **2021**, *20* (4), 1864–1873. https://doi.org/10.1021/acs.jproteome.0c00863.
